# Glucose Enhances Salinity-Driven Sea Spray Aerosol Production in Eastern Arctic Waters

**DOI:** 10.1021/acs.est.4c02826

**Published:** 2024-05-06

**Authors:** Arianna Rocchi, Anabel von Jackowski, André Welti, Guangyu Li, Zamin A. Kanji, Vasiliy Povazhnyy, Anja Engel, Julia Schmale, Athanasios Nenes, Elisa Berdalet, Rafel Simó, Manuel Dall′Osto

**Affiliations:** † Department of Marine Biology and Oceanography, Institute of Marine Sciences (ICM, CSIC), Pg. Marítim de la Barceloneta, 37-49, E-08003 Barcelona, Spain; ‡ Faculty of Earth Sciences, University of Barcelona, Carrer Martí i Franquès, s/n, E-08028 Barcelona, Spain; § GEOMAR Helmholtz Centre for Ocean Research Kiel, Wischhofstraße 1−3, 24148 Kiel, Germany; ∥ Finnish Meteorological Institute, Erik Palménin aukio, 1. 00560 Helsinki, Finland; ⊥ Institute for Atmospheric and Climate Science, ETH Zurich, Universitätstrasse 16, 8092 Zurich, Switzerland; # The Otto Schmidt Laboratory, Arctic and Antarctic Research Institute, Beringa, 38. 199397 St. Petersburg, Russia; ∇ École Polytechnique Fédérale de Lausanne, EPFL, CH-1015 Lausanne, Switzerland

**Keywords:** marine biogeochemistry, Arctic, polar aerosol production, aerosol chamber, organic matter, climate change

## Abstract

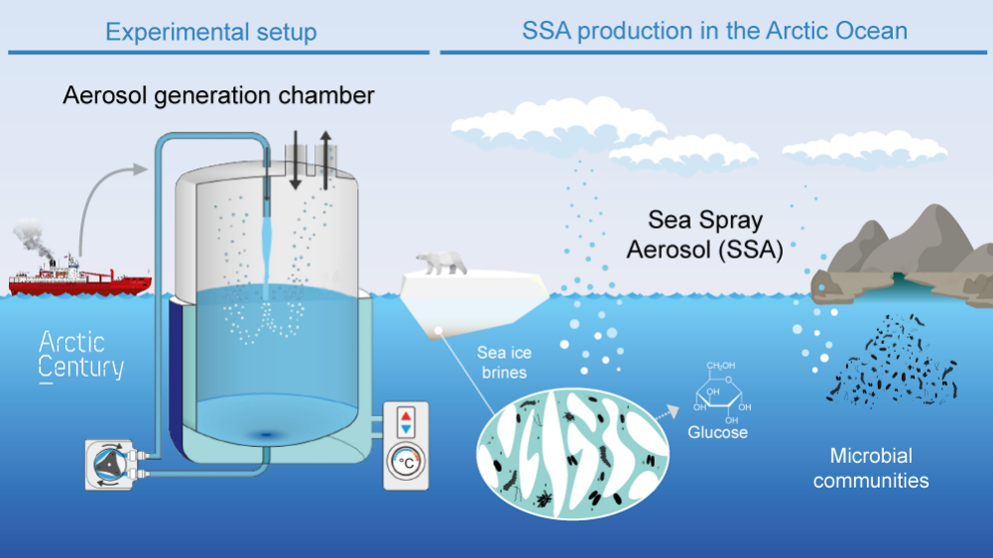

Sea spray aerosols (SSA) greatly affect the climate system by scattering solar radiation and acting as seeds for cloud droplet formation. The ecosystems in the Arctic Ocean are rapidly changing due to global warming, and the effects these changes have on the generation of SSA, and thereby clouds and fog formation in this region, are unknown. During the ship-based Arctic Century Expedition, we examined the dependency of forced SSA production on the biogeochemical characteristics of seawater using an on-board temperature-controlled aerosol generation chamber with a plunging jet system. Our results indicate that mainly seawater salinity and organic content influence the production and size distribution of SSA. However, we observed a 2-fold higher SSA production from waters with similar salinity collected north of 81°N compared to samples collected south of this latitude. This variability was not explained by phytoplankton and bacterial abundances or Chlorophyll-a concentration but by the presence of glucose in seawater. The synergic action of sea salt (essential component) and glucose or glucose-rich saccharides (enhancer) accounts for >80% of SSA predictability throughout the cruise. Our results suggest that besides wind speed and salinity, SSA production in Arctic waters is also affected by specific organics released by the microbiota.

## Abstract 2

This study identifies sea surface salinity and organics (particularly glucose) as key synergistic drivers of sea spray aerosol production in Arctic waters with a range of proximities to the sea-ice edge and riverine runoff.

## Introduction

In the last decades, the temperature increase in the Arctic has caused a rapid decline of the sea-ice extent and thickness, as well as an increase in river water input and precipitation^1−5^ with subsequent dramatic changes that affect not only the local environment but also the global climate system. Changes in the Arctic Ocean (AO) have cascading effects from ocean circulation and mixing to regional weather patterns and radiative balance and climate conditions worldwide. Therefore, a better understanding of ocean–atmosphere interactions in the Arctic is essential for predicting and mitigating global warming impacts. The AO surface water is becoming warmer and fresher with expanding open sea areas delimited by ice or land. This leads to as yet poorly understood modifications in the biogeochemical and microbial composition of AO waters as well as a potential increase in aerosol production.^6−9^ The AO is characterized by a large hydrographic heterogeneity caused by the influence of freshwater sources from ice cap melt and riverine runoff, and the impact of sea-ice formation and melt. This results in a broad range of sea surface salinities, which are more acute during the boreal summer. Due to this hydrographic heterogeneity, sea spray aerosol (SSA) production in the AO is expected to differ from that in the lower-latitude open oceans.^10^ Brean et al.^11^ demonstrated that the geographic location of Arctic surface waters emitting aerosol precursors results in an additional source of variability on secondary aerosol production. Whether a similar variability occurs with sea spray primary aerosols remains unknown.

Ocean waves, splashes, whirls, turbulences interacting with sea-ice edges and floes, and bubbles generated by processes beneath the ocean surface release SSA by bubble bursting into film and jet drops.^3,12−14^ SSA plays an important role in the climate system through direct and indirect radiative effects in the atmosphere.^15^ In particular, SSA can absorb water and serve as cloud condensation nuclei (CCN).^16,17^ The concentration of CCN influences the number and size of forming droplets, affecting cloud brightness, lifetime, and precipitation.^18^ Moreover, some SSA can act as ice nucleating particles, which facilitate ice crystal formation in mixed-phase clouds.^19,20^ The constituents of SSA, which are generally a mixture of inorganic salts and organic matter,^6,21^ are mostly determined by the water biogeochemistry.

Several studies in the Arctic indicated that inorganic salts are a driving factor in the production of SSA.^22−24^ Harb and Foroutan^25^ demonstrated that salinity enhances bubble-bursting mechanisms and the resulting sea spray ejection. Other investigations highlighted additional key factors for SSA production, such as sea surface temperature, upper ocean turbulence, seawater density, and concentration of organics. These factors affect the seawater surface tension, rise time of bubbles, and the bubble-bursting processes, and consequently, also affect sea spray ejection.^21,26−30^ Park et al.,^31^ for instance, provided evidence that SSA production is enhanced by riverine organics. The AO surface waters harbor microorganisms and a myriad of active organic compounds such as proteins, carbohydrates (saccharides), fatty acids, amino acids, exopolymers, and cellular debris.^10,32−37^ Most of these compounds are planktonic food web byproducts and small molecular weight metabolites that are released as phytoplankton exudates.^38^ Many of these organic compounds, mixed up with sea salt, are released as SSA into the atmosphere through bubble bursting.^39^ It has been shown that the organic fraction of SSA is characterized by molecular signatures of saccharides (up to 61% of the submicron SSA organic mass) and carboxylic acids.^40−42^ More recently, Zeppenfeld et al.^43^ found a high enrichment factor of small carbohydrates (glucose, fructose, rhamnose, and glucosamine) in supermicron and mainly submicron particles relative to the seawater fraction in the Antarctic Peninsula and in the Arctic aerosol and fog.^44^ However, the relative contributions of inorganic salts and organic matter (OM) on the production of primary marine aerosols are still under debate.

The composition of OM in AO surface waters depends on multiple factors, both allochthonous and autochthonous. Riverine and glacier discharges transport terrestrial OM but also inorganic particles and micro- and macro-nutrients.^45^ An increase in riverine outflows is causing documented changes in the biological marine communities.^45,46^ Furthermore, sea ice is full of biological activity, as abundant populations of viruses, prokaryotic microbes, and protists live inside brine channels.^47^ When sea ice starts to melt in early summer, the inhabiting microorganisms and their organic metabolites and debris are released into the seawater. Thus, SSA production and composition are expected to be affected by OM derived from the ocean and sea-ice microbiota and continental runoff. The organic/inorganic composition of SSA was modeled in Burrows et al.^48−50^ through deepening the relationships between SSA chemistry and ocean biogeochemistry.

Among the diversity of OM components that may be critical for SSA production in the Arctic, carbohydrates stand out as potential candidates. They have been identified in Arctic aerosols over the last 10 years.^51^ Before that, in the early 2000s, Herborg et al.^52^ and Underwood et al.^53^ identified glucose as an abundant component in sea-ice environments and highlighted the role of sea ice as a source of dissolved carbohydrates in seawater. Recently, Zeppenfeld et al.^44^ also highlighted the importance of sea ice in determining the distribution of carbohydrates in Arctic waters. All in all, the expectation is that the impact of organics on SSA production will be particularly high in waters under the influence of sea-ice melt.^54,55^

The present study aims to increase our understanding of the main drivers of SSA formation in the Arctic. During the Arctic Century expedition to the eastern Arctic Ocean in late summer 2021, we conducted 18 experiments with an on-board aerosol generation chamber, with the aim to characterize the number concentration and size distribution of SSA forcedly ejected from surface seawater and compare them with the seawater biogeochemical composition, with a particular focus on certain carbohydrates/sugars. The distribution of the experiments along the cruise track covered a latitudinal and hydrographic transect characterized by distinct proximity to riverine discharges and the sea-ice edge, resulting in distinct salinity and OM composition. Statistical relationships between SSA number concentrations in several size ranges and the salinity and concentrations of seawater components shed light on the role of biogenic organic matter in enhancing salinity-driven SSA formation in the Arctic.

## Materials and Methods

### Study Area, Water Sampling Strategy, and Experiment Design

**Figure 1:**
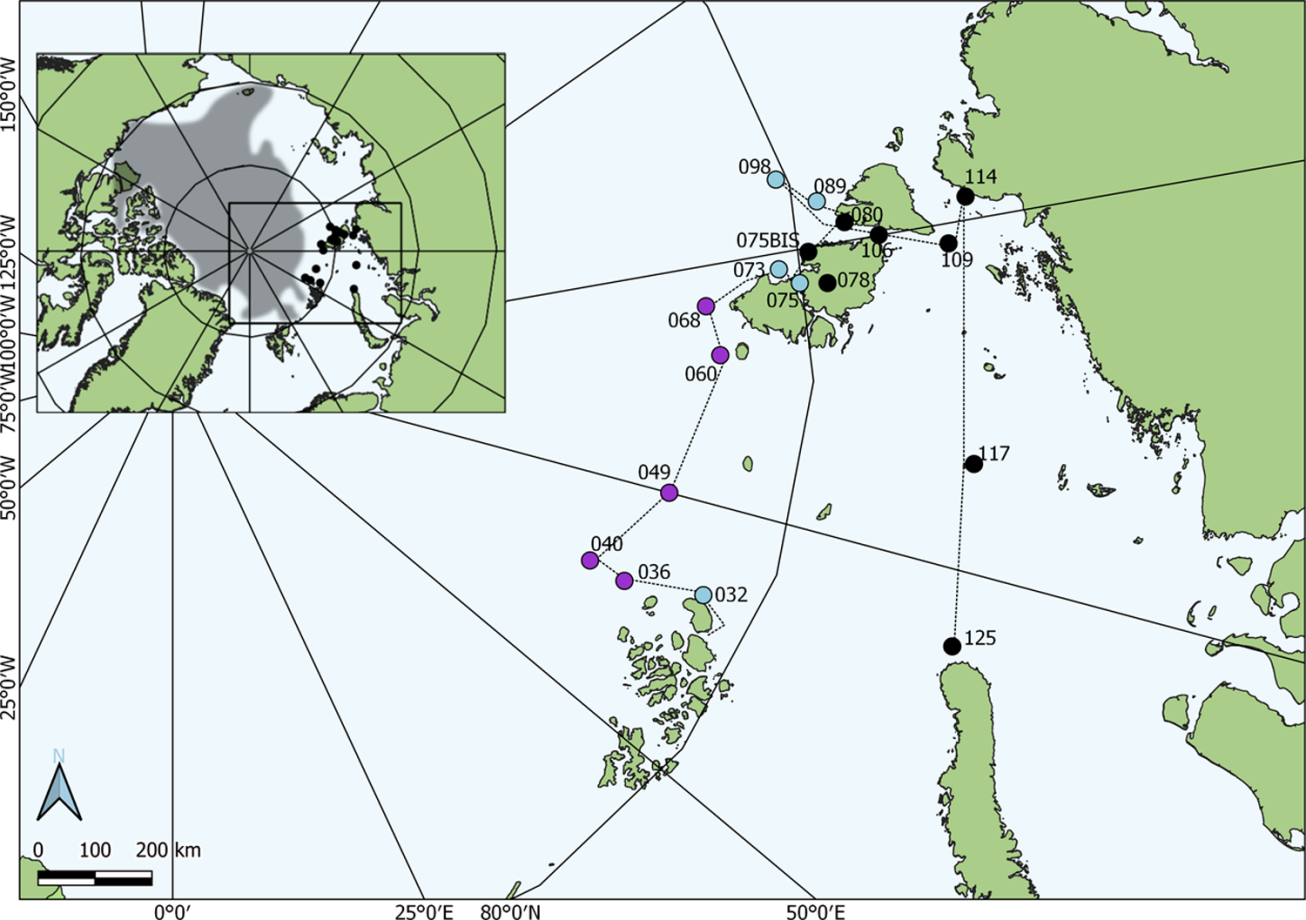
Map of the Russian Arctic (inset) showing the transect of the Arctic Century Expedition and the water sampling sites. The scientific cruise path aboard the Research Vessel Akademik Tryoshnikov is indicated with a dotted line; filled symbols (*n* = 18) indicate the stations where water was sampled. To facilitate location, purple symbols indicate those collected North of the 81°N latitude (*n* = 5); blue symbols indicate those obtained between 79°N and 81°N (*n* = 5); and black symbols indicate the rest of samples (*n* = 8). The coordinates are indicated in Supporting Table S1. The gray area (in the inset map) indicates the extent of the sea ice in August 2021, based on Copernicus and ECMWF satellite images.

The Arctic Century expedition was conducted in the eastern Arctic aboard the Russian Research Vessel Akademik Tryoshnikov. Water samples were collected in 18 water sampling sites (Figure 1 and Table S1) located south of 83°N, between 64°E and 116°E. This Arctic cruise allowed us to have the rare opportunity to visit and study the waters of the three Russian archipelagos engirdling the Kara Sea, influenced by melting glaciers, riverine runoff, sea-ice retreat, and permafrost thawing. A total of 15 seawater surface samples (32 L each) were obtained at ca. 2 m depth using a SEA-BIRD conductivity–temperature–depth (CTD) rosette sampling system equipped with 24 Niskin bottles. Seawater temperature (°C), fluorescence (mg m^–3^), and salinity (‰, grams of salt per kilogram of water) were measured by the CTD probes during downcasts (Table S1). In addition, three samples (#075BIS, #078, and #080) were collected using a plastic bucket on shore of Pioneer Island, October Revolution Island Lake and Cape Baranov, respectively. To obtain low-intermediate salinity water, 12 L of the water collected at station #075BIS was mixed with 20 L of a previous sample (#075), which had been conserved on board at ca. −1 °C (corresponding to the in situ temperature, Table S1). From each water sample, 30 L was used for the experiments in the aerosol generation chamber and 2 L for the characterization of biogeochemical variables in the water prior to each experiment, representing the biogeochemical properties of the surface water. Additional samples were collected at the end of the bubbling period to control the potential changes during the incubation.

### Aerosol Generation Chamber and Experimental Setup

All water samples were incubated in the aerosol generation chamber immediately after collection. Details of the chamber are provided in previous studies conducted in Antarctica,^31,56^ and in the Mediterranean Sea.^57^ We are aware that laboratory experiments cannot fully reproduce real ocean conditions; however, they play a valuable role in revealing the fundamental mechanisms involved in ocean-atmosphere processes. Our objective with the aerosol-generating chamber is to isolate and study the SSA coming from different seawater from the background air. Briefly, the chamber consists of a 60 L airtight high-grade stainless-steel cylinder (60 cm height, 41 cm diameter, internal dimensions) with a temperature-controlled jacket to allow maintaining the water temperature to prevent changes in aerosol production due to temperature changes^58^ during an experiment. Seawater from 6.5 m deep was pumped constantly and recirculated through the jacket, maintaining the water sample at −1.7 to +3.2 °C (Table S1).

Several ports allow recirculation of the incubated water (with a peristaltic pump) and the aerosols sampling. The plunging jet settings (water flow, distance to water surface) followed Fuentes et al.^26^ to reproduce experimentally generated bubble size distributions similar to those observed in the ocean. At the beginning of each experiment, filtered air was flushed through the chamber to remove any contamination from the laboratory air. Before and after each experiment, careful cleaning protocols were applied with several Milli-Q water rinsings. In addition, a successful control experiment was carried out with 30 L of Milli-Q water, in which no aerosol formation was observed.

### SSA Concentration and Size Distribution Measurement

The SSA size distribution was characterized using a scanning mobility particle sizer (SMPS, Model 3938, comprising a DMA Model 3082 and a CPC Model 3787, TSI, Inc.) and an optical particle counter (OPC, Model GT-526S, MetOne). The instruments were connected to the chamber through a port for sampling aerosol. Two silica gel diffusion dryers in series were used to dry the particles before entering the SMPS and the OPC. The SMPS estimated the number of SSA particles across the 20–600 nm size range, classified in 110 size categories, with a 4 min scanning time. Multiple charge correction was applied to account for the misclassification of large particles with multiple charges. The OPC measured the aerosols on six binned, optical diameter fractions (i.e., >0.3, >0.5, >1, >2.5, >5, and >10 μm).

### Biogeochemical Variables

Phytoplankton (<50 μm) and bacterial abundances were counted with a flow cytometer (FACSCalibur, Becton Dickinson). To account for the upper detection limit of the FACSCalibur, samples were prefiltered through a 50 μm mesh. Seawater subsamples were fixed with glutaraldehyde (2% final concentration), incubated for 15 min at room temperature, and then frozen at −80 °C until analysis at GEOMAR. The autotrophic cells were counted using orange autofluorescence (*Synechococcus* < 2 μm and cryptophytes) and red fluorescence (picoeukaryotes <2 μm and nanoplankton ∼2 to 20 μm).^59^ The heterotrophic cells were incubated in the dark with Sybr Green (Invitrogen) for 5 min and, once injected, counted by detecting the DNA-binding dye in the cytogram using the software CellQuest Pro (Becton Dickinson).

Total Chlorophyll-a (Chl-a) (μg L^–1^) concentration, a proxy of the total photosynthetic biomass, was estimated on 1 L seawater samples filtered through 25 mm, grade GF/F filters (Whatman). Chl-a was extracted from filters and immediately processed on-board. Chl-a extraction was performed on 90% acetone for 24 h in dark conditions. Extracts were read on a Turner Designs Trilogy fluorometer calibrated with pure spinach Chl-a (Sigma, C5753).

The following biochemical variables were measured on 1 L of water previously filtered through a 10 μm polycarbonate filter. A sample (20 mL) for simultaneous estimation of dissolved organic carbon (DOC) and total dissolved nitrogen (TDN) was filtered through 0.45 μm glass microfiber filters (GMF, grade GD/X, Whatman, U.K.) and collected into combusted glass ampules (450 °C, 8 h). The sample was acidified with 4 M hydrochloric acid, flame-sealed, and stored at 4 °C until analysis at GEOMAR. DOC/TDN were analyzed using high-temperature catalytic oxidation (TOC-VCSH, Shimadzu) with a detection limit of 1 μmol L^–1^.^60,61^ The concentration of dissolved organic nitrogen (DON) was estimated from the subtraction of the sum of nitrate and nitrite from TDN. High-molecular-weight (>1 kDa) dissolved combined carbohydrates (DCCHO) were sampled by filtering 20 mL of seawater through 0.45 μm Acrodisk filters (Pall), collected in combusted glass vials (450 °C, 8 h), and frozen (−20 °C) until analysis at GEOMAR. DCCHO were quantified using high-performance anion exchange chromatography coupled with pulsed amperometric detection (HPAEC-PAD, ICS 3000, Dionex) with a CarboPac PA10 analytical column (Dionex).^62^ HPAEC-PAD analysis classified individual monomers: arabinose, fucose, galactose, galactosamine, galacturonic acid, glucose, glucosamine, glucuronic, rhamnose, coelute mannose, and xylose.

## Results and Discussion

### Impacts of Salinity and Latitude on SSA Production

The water samples were collected across a wide salinity range (Table S1). The sample collected in October Revolution Island Lake (no. 078) was freshwater (0 salinity); sample nos. 109 and 114 were obtained in brackish waters with low to intermediate salinity values (ca. 14.5), while sample nos. 080, 106, 117, and 125 with salinities close to 30 were in the transition from brackish to marine waters category. The 10 remaining samples presented a narrow range of seawater salinities (32.1–33.5, 33 ± 1). The temperature of most samples was between −1.6 and +1.7 °C, with the highest register in the brackish and brackish–marine transition waters of the southernmost sites (1.8–3.2 °C) (Table S1). Note that the freshwater, brackish, and brackish–marine transition water samples were obtained at latitudes south of 80°N and the more saline ones were collected above (or very close to) that latitude (Figure 1 and Table S1). During our cruise, the sea-ice extent was markedly reduced to 5.6 million km^2^ compared to 7.2 million km^2^ for the 1981–2010 average. Satellite images provided by Copernicus and the European Centre for Medium-Range Weather Forecasts (ECMWF) indicate a negative sea-ice anomaly of 25–75% in the northern Kara Sea during August 2021. This situation may have allowed Atlantic waters to enter that part of the Arctic basin.^45^ In contrast, at stations #109 and #114 where the salinity was 14.4 and 14.6, respectively, a 25% positive sea-ice anomaly was observed. As known, the salinity range, the main factor determining seawater density, has a significant impact on seawater mixing and circulation in the AO.^45^

**Figure 2:**
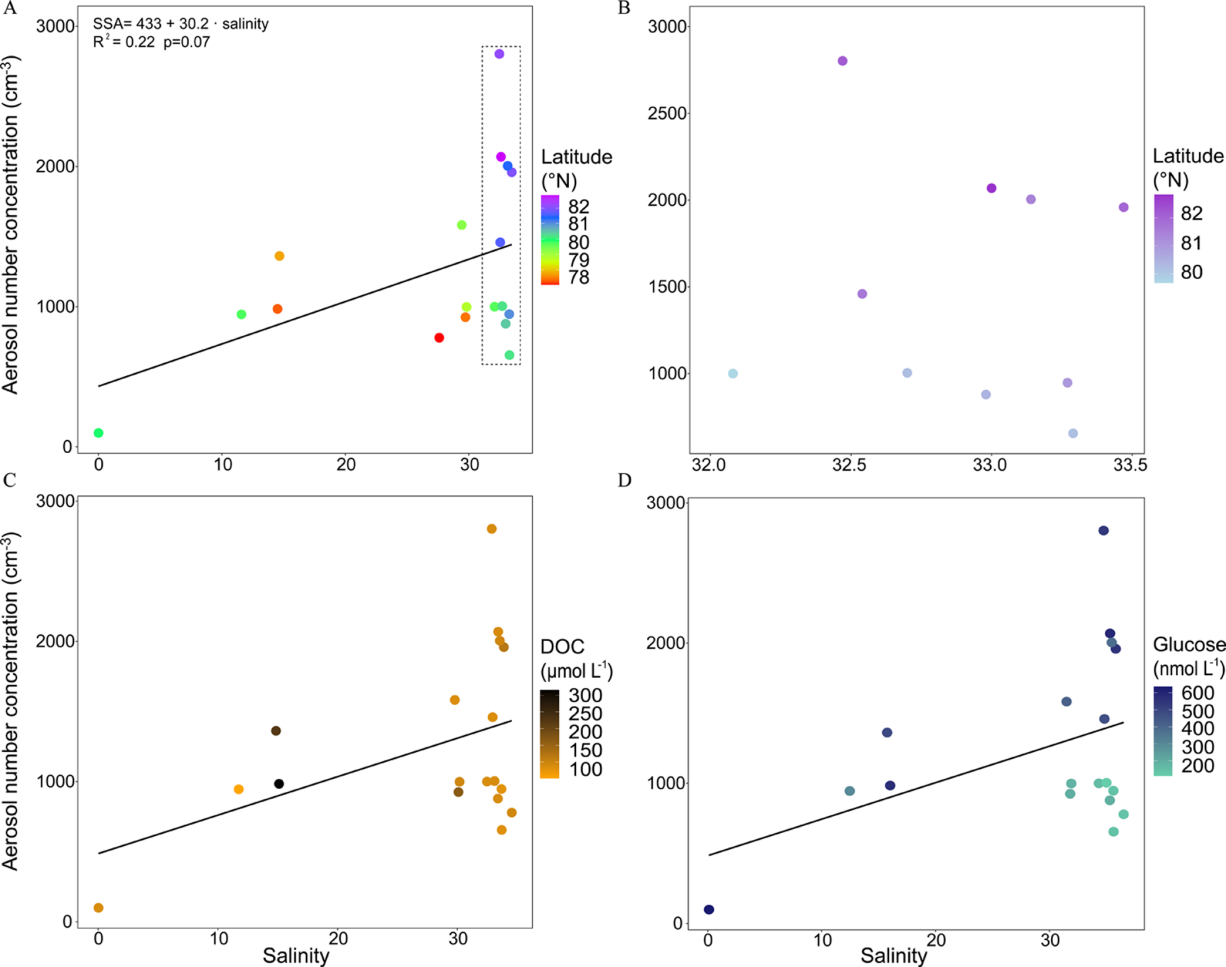
Scatter plots of the concentration of SSA (cm^–3^) versus salinity using a color scale for latitude showing all data ((A) *n* = 18) and the subset of the samples with salinity 33 ± 1 ((B) *n* = 10) corresponding to the inset in (A). The DOC and glucose concentrations in the 18 stations are presented in (C) and (D), respectively, using color scale gradients. The regression line between SSA versus salinity is indicated in (A, C, D), which is given by SSA = 433 + 30.2 salinity, *R*^2^ = 0.22, *p* = 0.07 for all three panels.

Figure 2A shows the produced SSA number concentration (cm^–3^) as a function of the salinity. The correlation between the SSA concentration and salinity was positive but poor (*R*^2^ = 0.22). However, the lake water sample with an aerosol production almost null (99 cm^–3^) corroborates that in the absence of inorganic salts, primary aerosol ejection from bubble bursting is substantially limited.^24^ Aerosol production was higher (984–1360 cm^–3^) from incubated brackish waters (salinity 11.6–14.6). In this category, the experiment with the artificially generated salinity of 11.6 is also included (#075BIS, Table S1). At higher salinities (27.7–33.5), comprising the brackish–marine transition waters, a wide range of produced aerosol concentrations (655–2803 cm^–3^) were measured, overlapping with the concentrations from the brackish water samples. In experiments using artificial saline waters at 20 °C, Zinke et al.^30^ found a maximum in SSA production at intermediate (5–10) salinities. Sofieva et al.^63^ observed a modest dependence of aerosol production on water salinity, but a strong dependence on temperature below 10 °C. However, in our field experiments, sea surface temperature did not further explain SSA production trends, likely because the experiments were conducted within a narrow temperature range.^27^

Overall, the experiments confirm that salinity is fundamental for SSA production, as found in previous studies conducted on various latitudes,^64−66^ including Arctic waters.^67,68^ Nonetheless, the poor correlation and the large variation of SSA concentration generated from waters within a narrow salinity range (33 ± 1) indicate that the concentration of the produced SSA was not uniquely linked to salinity. Considering the higher-salinity waters only, two groups of samples are distinguished according to latitude (Figure 2B):

(i) 5 samples collected north of 81°N, producing high SSA concentrations (2059 ± 430 cm^–3^);
(ii) 5 samples obtained south of 81°N, with lower SSA production (897 ± 129 cm^–3^).

The observed link of the SSA aerosol production with latitude in the Arctic Century cruise could be linked in turn to the influence of sea-ice retreat: the samples obtained north of 81°N, in saline open sea waters, were likely affected by the Arctic Boundary Current after sea-ice melt occurred at the beginning of the season,^6^ while those obtained south of 81°N, corresponded to coastal waters less influenced by recent sea-ice melt.

### SSA Size Distribution

**Figure 3:**
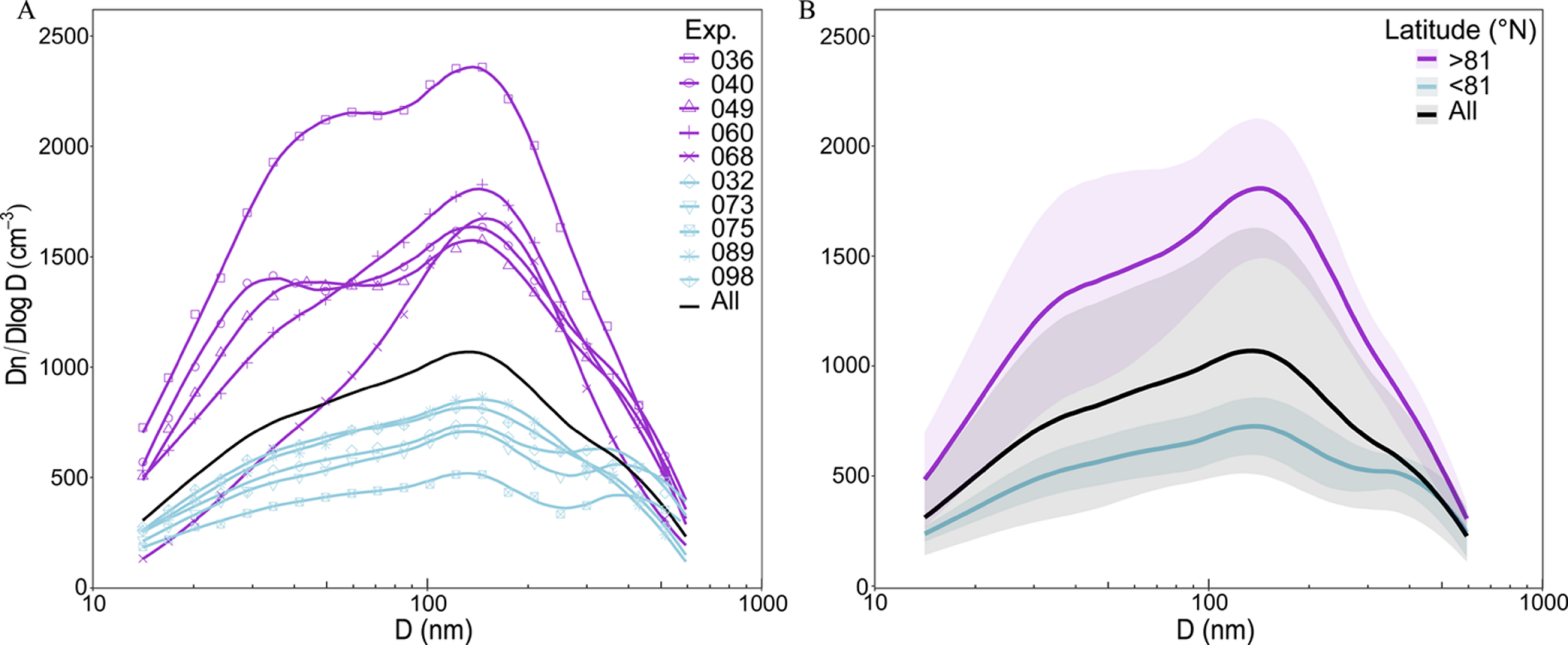
Aerosol size-resolved concentrations (cm^–3^) of the two latitudinal groups of samples during the aerosol-generating chamber experiments. (A) Size spectra of each experiment conducted with the 10 seawater samples with 33 ± 1 salinity; purple lines: samples collected above 81°N (>81); light blue: samples collected south of 81°N (≤81N). (B) Average of the two latitudinal groups of samples with their respective standard deviation, (*n* = 5, both groups). In the two graphs, the black line shows the average spectra considering all of the 18 samples, i.e., including freshwater, brackish, and brackish–marine transition waters.

The size spectra of the primary aerosol produced in the 18 experiments were very diverse (Figure S1). Focusing on the 10 more saline waters, the samples obtained north or south of 81°N (Figure 3) provided two different SSA patterns. A main size mode close to 160 nm (specific diameter of the SSA particles) and secondary modes around 30 and 350 nm were observed. The main size mode agrees with the study of Prather et al.^69^ who demonstrated that plunging jets generate SSA with a size distribution similar to that produced by breaking waves, with a mode around 162 ± 21 nm. All of the experiments also showed the 30 and 350 nm modes (Figure S1). Sellegri et al.^70^ and Fuentes et al.^26^ found the 300 nm mode in air-forced bubble bursting chamber experiments with seawater samples from the Mediterranean Sea and around New Zealand. The 30 nm mode was also found in other studies in several regions^68,70,71^ including the Arctic.^72^ We hypothesize that these observed modes are related to the composition of the particles, as discussed later.

### Influence of Biogeochemistry on SSA Production

To investigate the potential role of seawater microbiology on the SSA production, we analyzed the concentrations of Chl-a and the abundances of the main microbial groups: total bacteria, eukaryotic picoplankton smaller than 2 μm, eukaryotic nanoplankton in the size range ∼2 to 20 μm, and the main photosynthetic taxa, namely, cyanobacteria—*Synechococcus*— and protistan phytoplankton in the <50 μm size fraction, including cryptophytes. Comparisons were conducted between seawater samples in the two latitudinal groups, i.e., north or south of 81°N, that exhibited different SSA production (Table 1 and Figure S2).

**Table 1:** Average and Standard Deviation of the Microbial and Biochemical Variables Estimated in All Water Samples (*n* = 18) and Specifically, in the Two Groups of Marine Waters Determined by the Latitude of the Sampled Stations, i.e., North and South of 81°N (*n* = 5 in Both Cases)^a^

variables	all (*n* = 18)	north of 81°N (*n* = 5)	south of 81°N (*n* = 5)	*p*
microbial biomass indicators				
chlorophyll-a (μg L^–1^)	1.0 ± 1.1	1.4 ± 1.7	0.7 ± 0.3	0.84
bacteria (10^5^ cells mL^–1^)	7.2 ± 4.6	6.6 ± 2.8	11.2 ± 4.6	0.31
picophytoplankton (10^3^ cells mL^–1^)	8.0 ± 5.5	7.0 ± 7.0	10.1 ± 5.2	0.31
nanoplankton (10^3^ cells mL^–1^)*	3.7 ± 4.5	0.7 ± 0.4	5.8 ± 5.2	**0.016**
*Synechococcus* (10^4^ cells mL^–1^)	1.0 ± 1.6	0.4 ± 0.7	1.9 ± 2.4	0.31
biogeochemical				
DOC (μmol L^–1^)	112.5 ± 61.6	96.4 ± 12.9	84.6 ± 4.0	0.15
DON (μmol L^–1^)	6.7 ± 1.9	7.4 ± 1.4	6.1 ± 0.6	0.31
combined carbohydrates				
DCCHO (nmol L^–1^)	1066.8 ± 707.7	988.4 ± 166.9	711.1 ± 422.4	0.15
DCCHO-C (nmol L^–1^)	6207.4 ± 4109.1	5802.6 ± 975.2	4079.1 ± 2343.6	0.15
neutral sugars (nmol L^–1^)	977.2 ± 648.1	918.9 ± 161.8	650.2 ± 415.2	0.15
-glucose (nmol L^–1^)**	345.3 ± 185.5	508.7 ± 75.9	147.3 ± 26.0	**0.0079**
-rhamnose (nmol L^–1^)	64.6 ± 80.0	41.7 ± 13.7	24.1 ± 7.1	0.095
-arabinose (nmol L^–1^)	22.7 ± 30.2	13.6 ± 14.5	5.7 ± 2.1	0.31
galactosamine (nmol L^–1^)	3.1 ± 1.9	3.0 ± 0.5	2.6 ± 0.6	0.31

a *p* indicates the degree of significance (non-parametric Wilcoxon test, 68% of confidence) of the differences between the two groups; (*, **) significant differences (95 and 99% of confidence, respectively).

#### Chlorophyll-a and Microorganisms

The Chl-a concentrations (a proxy of phytoplankton biomass) measured in the two latitudinal groups of samples, 1.43 ± 1.66 μg L^–1^ (*n* = 5) north of 81°N and 0.72 ± 0.28 μg L^–1^ (*n* = 5) south of 81°N (Table 1), were not significantly different (Table 1 and Figure S2A) or correlated to SSA production. Recently, experiments conducted under the OCEANFILMS project and model, demonstrated that the concentration of Chl-a is not a direct predictor of the organic matter fraction in SSA.^10,50^ Other studies suggested that a time lag of 8–10 days must be considered (combined with wind speed) to predict the organic fraction of SSA from Chl-a concentrations in the surface ocean.^73,74^

The abundances of micronanoplankton (<50 μm) and bacteria decreased toward higher latitudes (Table 1 and Figure S2B–F), contrary to SSA production. These results are in agreement with Christiansen et al.,^75^ who did not observe a link between phytoplankton biomass and SSA emission fluxes, and with the suggestion that the abundances of marine plankton organisms have little impact on SSA postulated by Bates et al.^76^ However, in other studies, nanoplankton biomass was suggested to promote SSA production.^17,56^ This discrepancy between nanoplankton and SSA may be due to differences among the studied regions or the seasonal succession and physiological state of the encountered plankton communities.^77^ The production of SSA could be linked to the dissolved organic matter (DOM) associated with biological processes rather than to the abundances of the microorganisms themselves.^78^ Autochthonous marine dissolved organics come from exudates of living phytoplankton and heterotrophic organisms, as well as from senescing and dead cells.^38^ Therefore, the apparent inconsistency among studies suggests that the physiological state and the ecological succession of the microbial assemblages, whose characterization is a main unsolved challenge, may play an important role in contributing SSA production-enhancing organics.

#### Dissolved Organic Matter

**Figure 4:**
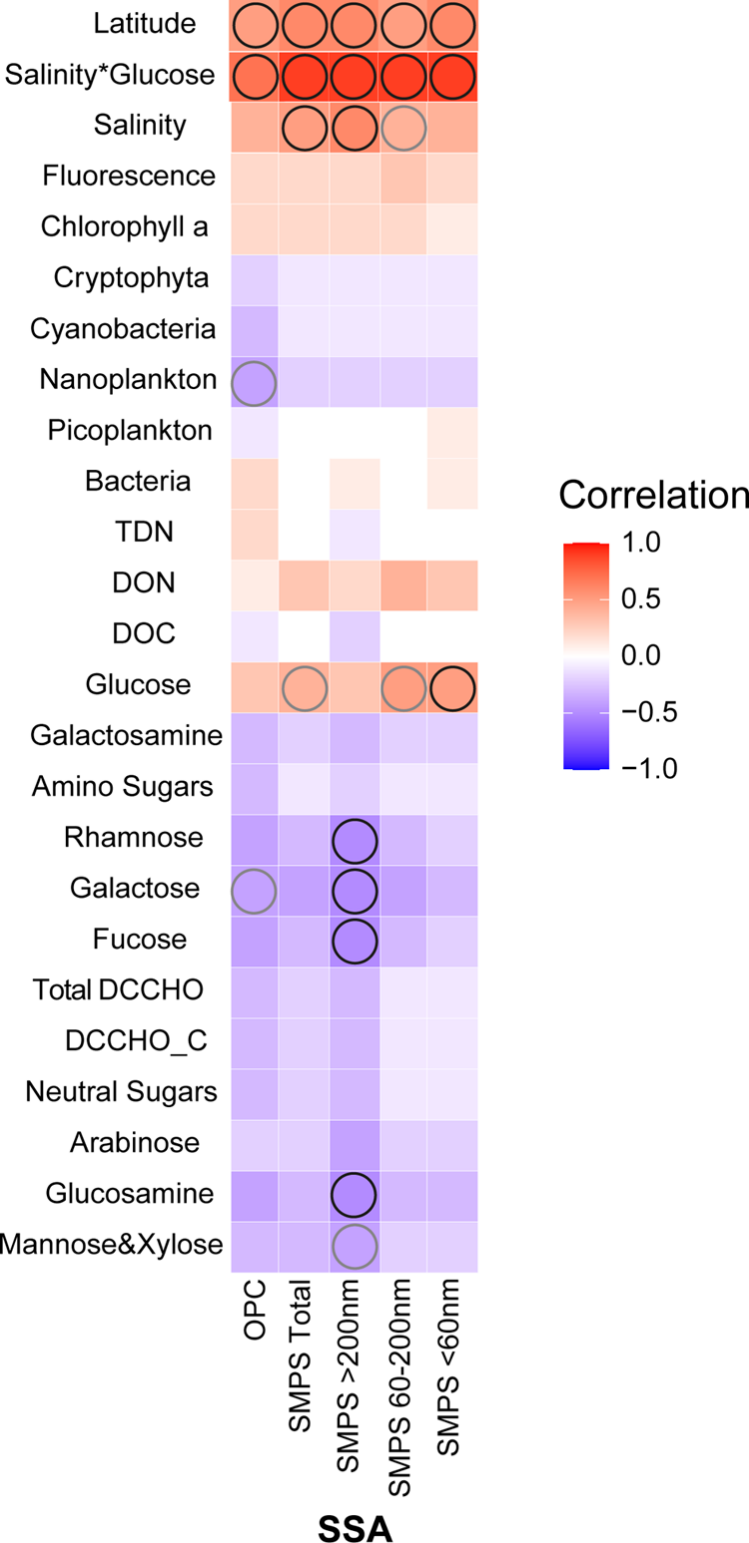
Heatmap showing Pearson’s correlations between size fractions of SMPS-measured SSA particles (<60, 60–200, >200 nm), OPC-measured SSA particles (300 nm–10 μm), and marine biogeochemical variables. The circles indicate significant correlations (black: *p* < 0.05; gray: *p* < 0.1). The number of samples for which data are correlated is 18.

In this study, DOM concentrations, including DOC and DON, were not significantly different in the two different latitude-based groups of samples (Table 1 and Figure S3A,B). A fraction of fresh DOC has biological origins from phytoplankton cells via physiological exudates, zooplankton grazing, and viral lysis.^79^ These processes release labile DOC that is bioavailable to heterotrophic microorganisms.^80,81^ More refractory DOC is typically derived from terrestrial sources.^82,83^ DOC is also released from sea-ice melt.^47,84^ Concerning organic nitrogen, in studies around the Antarctic Peninsula, Dall′Osto et al.^54,85^ and Rinaldi et al.^86^ suggested that DON can leak from melting sea ice and being incorporated into marine aerosols once transformed into methylamines, although the involved biological and chemical processes are poorly understood. However, we did not find any strong positive correlation between DOC or DON and SSA number concentration or aerosol size distribution (Table 1 and Figures 2C and 4), probably because the DOM components potentially active in changing air bubble properties and aerosol production were just a minor proportion of the total DOC and DON pools.

#### Saccharides

Concerning DCCHO (Table 1 and Figure S3), significant latitudinal differences were found in the following parameters: the concentrations of total carbohydrates (DCCHO, Figure S3C) and the carbon contained in carbohydrates (DCCHO-C, Figure S3D), thereof neutral sugars (Figure S3F), in particular glucose, rhamnose, and arabinose (Figure S3H,L,M, respectively). However, only glucose showed a significant positive correlation to SSA number concentrations (*r* = 0.73, *p* = 0.02, Figure 2D). This suggests that glucose is a major indicator of high SSA production in the Arctic.

#### Synergistic Impact of Glucose and Salinity on SSA Size Fractions

Furthermore, we assessed the cross-influence of glucose and salinity on the production of different SSA particle sizes (Figure 4). According to the measurement technique and the modal distributions observed, four main size groups of particles can be considered. There are four size groups measured with SMPS including (i) particles with diameters below 60 nm, which belong to the Aitken mode and are too small to be activated as CCN (Hoppel minimum); (ii) particles between 60 and 200 nm (smaller Accumulation mode); (iii) the largest particles (larger Accumulation mode) with diameters above 200 nm; and (iv) giant particles, mostly within the coarse mode, with diameters between 300 nm and 10 μm, measured by OPC. The smallest fraction of SSA, peaking at 30 nm, was strongly and positively correlated with glucose. The “intermediate” particles (60–200 nm) showed moderate positive correlation with both glucose and salinity. And the larger particles (>200 nm) were best correlated with salinity. All fractions (from either SMPS or OPC measurements) were positively correlated with the product of glucose and salinity. Actually, the combination of salinity and glucose explained 81% of the variance in total SSA (SSA number concentration = 0.1 salinity glucose + 415; *R*^2^ = 0.81; *n* = 18; *p* < 0.01). Therefore, the number concentration of total SSA generated could be predicted over 80% from two seawater variables. Our study suggests that inorganic salts and glucose acting synergically have a very strong regulating role for SSA generation in Arctic waters. Alpert et al.^87^ found evidence that phytoplankton exudates and their concomitant decomposition by bacteria act to enhance particle fluxes. The addition of algal exudates to seawater has been observed to reduce surface tension.^88^ They suggested that biological activity affects the amount and properties of seawater surfactants and consequently the surface tension of bubbles, which in turn alters bubble size and concentration and the subsequent SSA production.^87^ Common seawater surfactants, such as polysaccharides and glycolipids, have glucose moieties. Therefore, glucose may be the active agent to enhance SSA production or may indicate the occurrence of carbohydrate-rich surfactants of biological origin, which increase the bubble lifetime and SSA numbers. The interaction between glucose-containing surfactants and salt ions at the air–sea interface can alter the surface activity, composition, and properties of the resulting sea spray aerosols.^89^

#### Glucose in Arctic Sea Ice, Seawater, and Aerosols

In Arctic sea-ice, a high concentration of exopolymeric substances (EPS) produced by phytoplankton and constituted mainly of sugars (monosaccharides), polysaccharides, and proteins has been documented.^90−93^ Glucose in particular was identified as an important component of sea-ice habitats.^53^ About two decades ago, Herborg et al.^52^ pointed out sea-ice as a major source of dissolved carbohydrates. Since the main component of dissolved carbohydrates is glucose,^94^ freshly produced DOM in surface waters and sea ice was characterized by glucose proportions of up to 86%. Indeed, the composition of DOC exudates of sea-ice inhabiting microorganisms is dominated by glucose, which often constitutes more than 70% of the total.^95^ Recently, Piontek et al.^84^ confirmed that sea-ice polysaccharide composition is dominated by glucose (42–87 mol %). In accordance with that, in our study, seawater samples closer to the ice edge and influenced by sea-ice melt (stations above 81°N) contained more dissolved sugars, particularly glucose. Xu et al.^89^ observed that glucose additions led to an increase in particle number concentration in SSA generation experiments, as well as to a change in particle shape from prism-like to core–shell morphologies. Besides glucose, monosaccharides such as arabinose, galactose, mannose, and xylose have been detected in aerosols^51,96^ and ice nucleating particles^97^ in the Arctic. Similarly, Zeppenfeld et al.^43^ demonstrated that glucose and xylose dominated the monosaccharide composition of Antarctic SSA. Other studies found an enrichment of saccharides in SSA particles^6,41,98^ that were also enriched with bacteria,^99^ and most of the organic mass in Arctic primary aerosol emissions was classified as carbohydrate-like compounds.^10,40^ These Arctic studies add to ample evidence that SSA particles from diverse origins contain carbohydrates,^40,42,100−102^ in contrast to the initial OCEANFILMS model,^50^ in which polysaccharides were not substantially enriched at the air–water interface. Our study suggests that neutral sugars, particularly glucose, may facilitate SSA emissions during the Arctic summer and should be considered in modeling SSA. We hypothesize that small saccharides like glucose may be responsible for the 30 nm mode in the SSA size spectra of seawater samples closer to the sea-ice edge.

#### Environmental Relevance

Over the last century, the temperature in the Arctic region has risen more than twice the global average. Sea-ice melt impacts the marine biosphere by allowing a longer growing season in ocean waters. The annual net primary production has been reported to have increased by about 30% during the last decades.^103^ The cycle of freshwater in the Arctic has also undergone significant alterations. The annual freshwater discharge, containing terrestrial organics from the Eurasian rivers, has increased by 7%.^104^ Based on ecological criteria, 17 large Arctic marine ecosystems were defined in 2013 as susceptible to being substantially impacted by these undergoing changes in the Arctic region.^45^ Thus, SSA production could also be affected by these changes, with severe consequences for the ocean–atmosphere interactions in the Arctic.

In this work, the link between SSA production and the biogeochemistry of Arctic water samples was investigated. Our findings shed light on the ocean–atmosphere interactions in polar regions, which is valuable knowledge in the context of current warming of one of the most vulnerable areas of our planet. We identified three groups of samples, grouped by salinity and latitude and likely associated with distinct influences of freshwater discharges and sea-ice melt, that showed a clear difference in the production of SSA. The seawater variables that explained most of the variability in SSA production were salinity and glucose composition. We confirmed that salinity is an essential driving factor in SSA production so that below the 10–15 salinity threshold, SSA formation is largely suppressed. Above this threshold, the number concentrations of generated SSA varied with latitude, increasing with proximity to the ice edge and glucose concentration. Our results also showed how SSA production was favored by inorganic salts and glucose depending on particle size: larger SSA mainly correlated to salinity, while smaller SSA mainly correlated to glucose. The synergistic action of the two ingredients (expressed as the product of salinity and glucose concentration) accounted for more than 80% of the generated SSA variance, with implications for the potential number of CCN.

We showed that neither Chl-a nor microbial abundances are a suitable predictor of the SSA number concentrations, probably because the occurrence of surfactant-active substances like glucose is not simply proportional to phytoplankton abundance but depends on their physiological state, ecological succession, and food web structure. The chemical characterization of seawater components indicated that only specific organics enhance SSA production from bubble bursting. These specific organics were mainly exemplified by glucose, probably freshly released as such or as the main component of polysaccharides from sea-ice melt and surface ocean phytoplankton. Our results suggest that the 30 nm mode in the SSA size spectra was most affected by glucose/saccharides. All in all, our study conducted across contrasting biogeochemical conditions indicates that sea salts stand as the essential ingredient and saccharides as the enhancer for the ejection of SSA from the surface Eastern Arctic Ocean. Understanding the properties, composition, and sources of sea spray aerosols in the Arctic is important for accurately characterizing their role in climate models and better predicting warming in this climate-sensitive region. Our study contributes to exploring the controls on polar SSA production, but monitoring efforts that account for Arctic warming and freshening are needed to shed further light on the feedback between ocean–atmosphere interactions and the changing Arctic climate.

## Supporting Information Available

All water samples numbered chronologically, station names with their corresponding coordinates of collection, along with salinity and Chlorophyll-a concentrations (Table S1); aerosol size distribution spectra of the 18 samples and the Milli-Q test (Figure S1); and box plots comparing biogeochemical data analyzed between the two latitudinal groups (Figures S2 and S3) (PDF)
