# Supplementary material for: Glucose Enhances Salinity-Driven Sea Spray Aerosol Production in Eastern Arctic Waters
Source: Environ Sci Technol. 2024 May 6;58(20):8748–59. doi: 10.1021/acs.est.4c02826 (PMC11112759; doi:10.1021/acs.est.4c02826)
Supplement: Supplementary file 1 — es4c02826_si_001.pdf [file es4c02826_si_001.pdf]

SUPPORTING INFORMATION

Glucose enhances salinity-driven sea spray aerosol production in Eastern Arctic waters

Arianna Rocchi[1,2*], Anabel von Jackowski[3], André Welti[4], Guangyu Li[5], Zamin A. Kanji[5], Vasiliy Povazhnyy[6], Anja Engel[3], Julia Schmale[7], Athanasios Nenes[7], Elisa Berdalet[1], Rafel Simó[1] and Manuel Dall'Osto[1]

[1]Institute of Marine Sciences (ICM, CSIC), Department of Marine Biology and Oceanography, Pg. Marítim de la Barceloneta, 37-49. E-08003, Barcelona, Spain
[2]University of Barcelona, Faculty of Earth Sciences, Carrer Martí i Franquès, s/n. E-08028, Barcelona, Spain
[3]GEOMAR Helmholtz Centre for Ocean Research Kiel, Wischhofstraße 1 - 3, 24148 Kiel, Germany
[4]Finnish Meteorological Institute, Erik Palménin aukio, 1. 00560, Helsinki, Finland
[5]Institute for Atmospheric and Climate Science, ETH Zurich, Universitätstrasse 16, 8092 Zurich, Switzerland
[6]The Otto Schmidt Laboratory, Arctic and Antarctic Research Institute, Beringa, 38. 199397, St. Petersburg, Russia
[7]École Polytechnique Fédérale de Lausanne, EPFL, CH-1015 Lausanne, Switzerland

*Corresponding author rocchi@icm.csic.es

Summary

Number of pages: 6
Table: 1
Figures: 3

Table S1. The chronological order number of the water samples, the name of the stations and the coordinates where water was collected, salinity and Chlorophyll-a concentrations of the water samples.

N	STATION	COORDINATES	Salinity	Temperature (°C)	Chlorophyll-a (μg L^{-1})
1	**#032**	81 00.11 N, 65 71.47 E	33.3	1.7	0.2
2	**#036**	82 16.57 N, 64 34.15 E	32.5	-1.0	0.1
3	**#040**	82 83.29 N, 64 32.36 E	32.6	-1.4	0.9
4	**#049**	81 99.94 N, 75 01.46 E	33.5	1.4	0.6
5	**#060**	81 39.92 N, 92 09.93 E	33.1	-1.6	4.7
6	**#068**	81 63.44 N, 95 69.64 E	32.5	-1.5	0.9
7	**#073**	80 42.91 N, 98 01.09 E	33.0	-0.3	1.1
8	**#075**	80 17.35 N, 96 94.58 E	33.3	-1.0	0.7
9	**#075BIS**	79 43.80 N, 96 32.40 E	11.6	NA	0.1
10	**#078**	79 57.69 N, 99 24.30 E	0.0	NA	1.5
11	**#080**	79 28.40 N, 101 58.80 E	29.4	-1.0	1.5
12	**#089**	79 67.41 N, 103 84.13 E	32.1	0.8	0.9
13	**#098**	80 20.25 N, 106 83.73 E	32.7	0.4	0.8
14	**#106**	78 79.70 N, 99 99.98 E	29.8	-0.8	0.9
15	**#109**	77 76.00 N, 98 66.54 E	14.6	0.0	0.7
16	**#114**	77 28.87 N, 101 64.72 E	14.4	1.8	NA
17	**#117**	77 42.12 N, 82 54.41 E	29.7	3.2	NA
18	**#125**	77 02.97 N, 70 05.82 E	27.7	3.0	NA

NA: not available

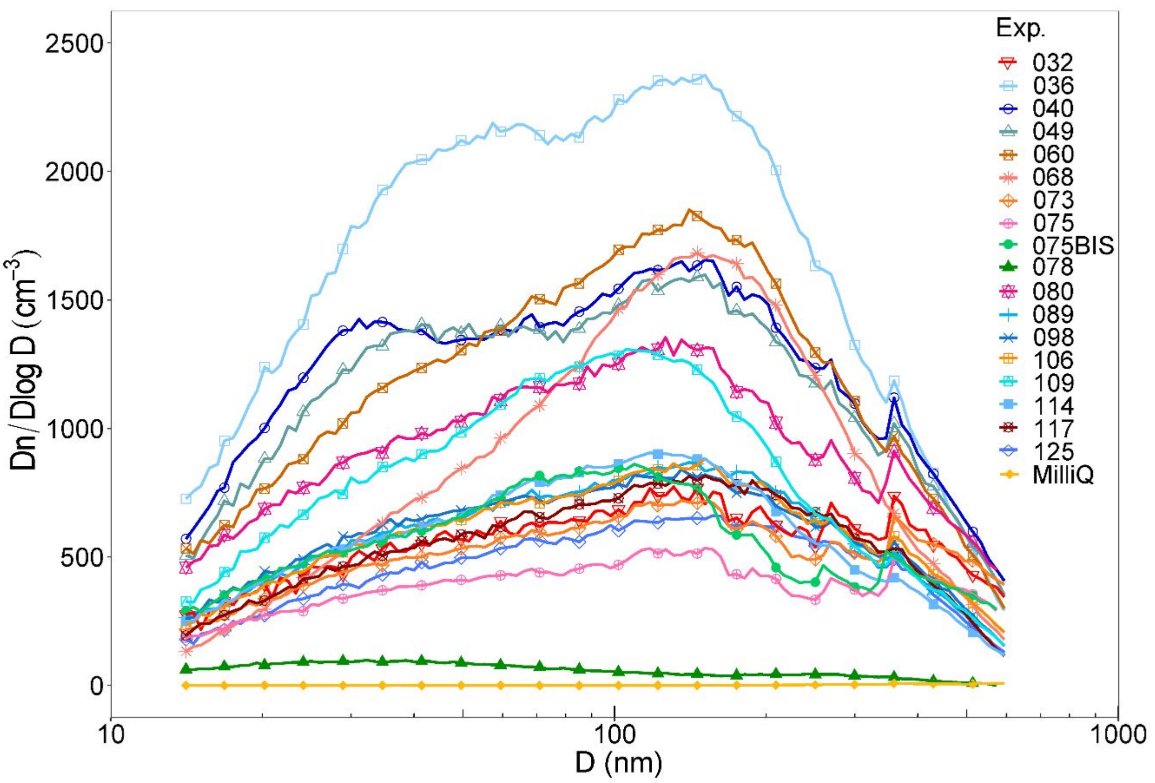

Figure S1. Aerosol size-resolved concentrations (cm^{-3}) of all the 18 samples and the MilliQ test (0 cm^{-3}).

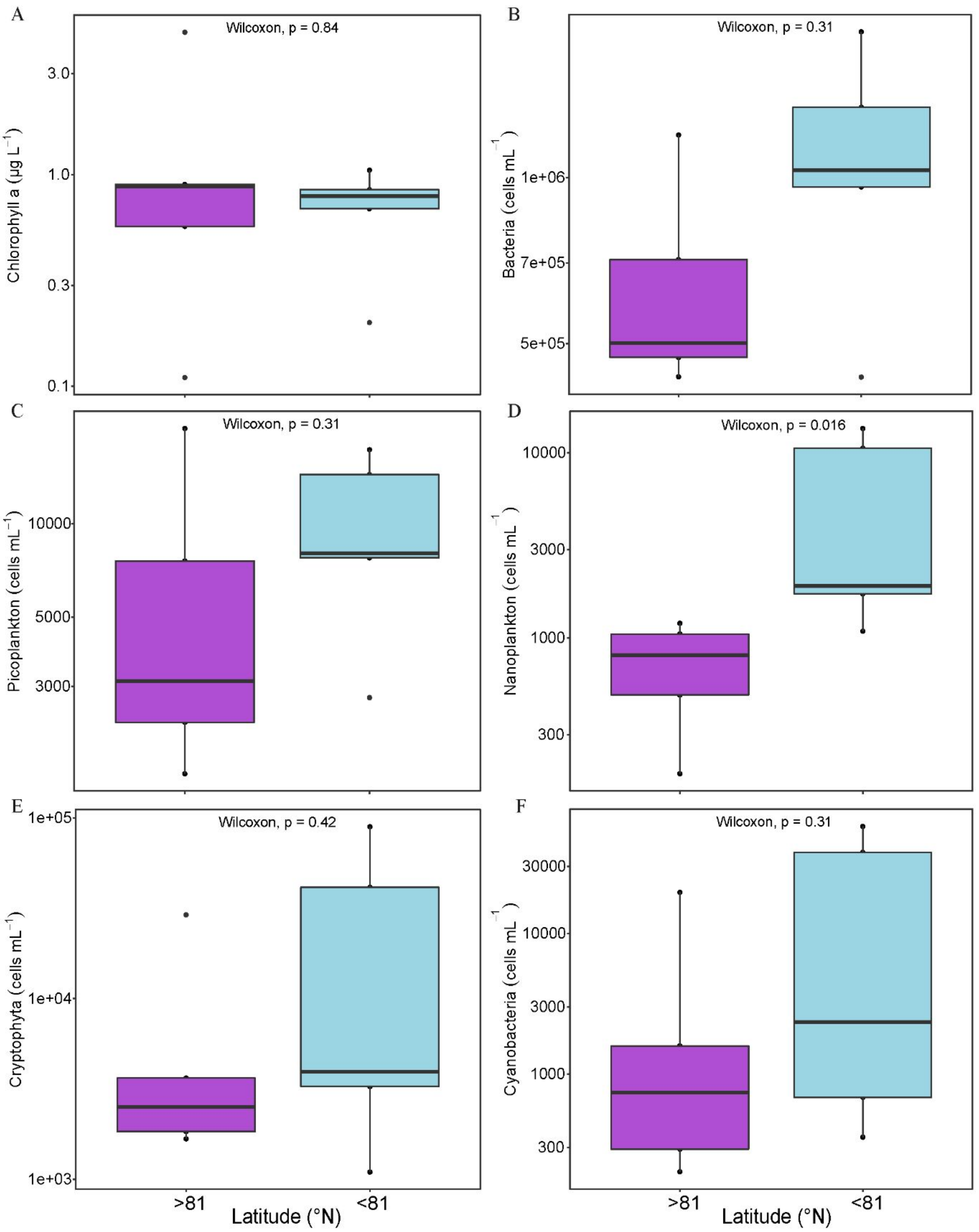

Figure S2. Box-plots of (A) Chlorophyll-a concentration, and cell abundances of (B) Bacteria, (C) Picoplankton (2 µm), (D) Nanoplankton (~2-20µm), (E) Cryptophyta and (F) Cyanobacteria (*Synechococcus*). Y-axes are in logarithmic scale. The error bars represent the standard deviation according to the number of samples (n=5 for both groups); horizontal lines within boxes indicate the median of the distribution and the dots represent the outliers.

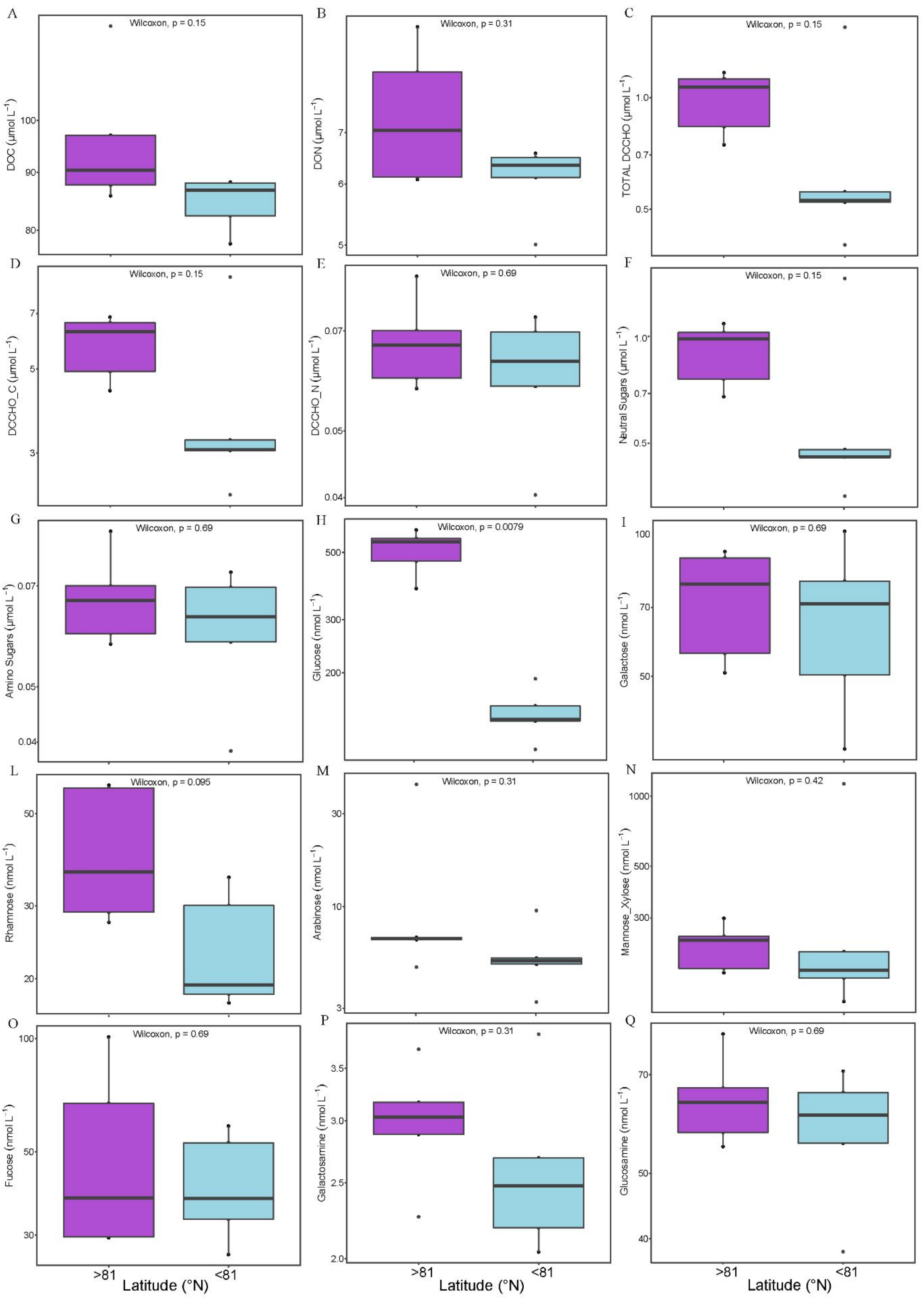

Figure S3. Box-plots of the concentrations of (A) Dissolved Organic Carbon (DOC), (B) Dissolved Organic Nitrogen (DON), (C) total Dissolved Combined Carbohydrates (DCCHO), Carbon and Nitrogen content in all

measured carbohydrates: (D) DCCHO_C and (E) DCCHO_N, (F) Neutral Sugars, (G) Amino Sugars, (H) Glucose, (I) Galactose, (L) Rhamnose, (M) Arabinose, (N) Mannose and Xylose, (O) Fucose, (P) Galactosamine and (Q) Glucosamine. Y-axes are in logarithmic scale. The error bars represent the standard deviation according to the number of samples (n=5 for both groups); horizontal lines within boxes indicate the median of the distribution and the dots represent the outliers.
